# Phenotype-guided selection of venlafaxine and topiramate for vestibular migraine: a narrative review

**DOI:** 10.3389/fnins.2026.1887023

**Published:** 2026-07-29

**Authors:** Tao Wang, Siwen Luo, Jingyang Han, Han Li, Yali Wang, Xiaotong Zhang, Dandan Li, Xinjun Yu

**Affiliations:** 1 Department of Geriatrics, Affiliated Hospital of Shandong Second Medical University, Weifang, China; 2 School of Clinical Medicine, Shandong Second Medical University, Weifang, China

**Keywords:** migraine prophylaxis, phenotype-guided treatment, topiramate, venlafaxine, vestibular migraine

## Abstract

Vestibular migraine (VM) is a common but underdiagnosed cause of recurrent vertigo. Its heterogeneous manifestations and the lack of validated biomarkers complicate diagnosis, and emerging evidence supports individualized rather than uniform preventive strategies. Among available prophylactic options, venlafaxine and topiramate are two clinically important agents with distinct mechanisms and therapeutic profiles. They are discussed here as illustrative exemplars rather than exclusive preferential choices. Venlafaxine may be a reasonable option to consider for patients with comorbid anxiety or depression, visually induced dizziness, persistent postural-perceptual dizziness (PPPD) overlap, or marked emotional and functional impairment. In contrast, topiramate may be more suitable for patients with a headache-predominant phenotype, pronounced sensory hypersensitivity, or a greater need for conventional migraine prophylaxis. Current studies suggest that both agents can reduce vestibular symptom burden; however, the overall quality of evidence remains limited by small sample sizes, heterogeneous diagnostic criteria, mixed inclusion of probable and definite VM, and inconsistent outcome measures. This review summarizes the current evidence comparing the two drugs, critically discusses their respective therapeutic roles, and proposes a pragmatic phenotype-guided framework for clinical decision-making. We emphasize that treatment selection should consider not only attack frequency and vertigo severity, but also psychiatric comorbidity, PPPD overlap, headache predominance, body-weight concerns, cognitive vulnerability, and functional recovery goals. Further phenotype-stratified prospective studies are needed to validate precision treatment strategies for VM.

## 1 Introduction

VM is now an established diagnostic entity at the interface of headache medicine, neuro-otology, and functional vestibular disorders (Lempert et al., 2022; Furman et al., 2013). The 2022 Bárány Society/International Headache Society update defines VM on the basis of recurrent vestibular symptoms of moderate or severe intensity, a current or previous history of migraine, a temporal relationship between vestibular episodes and migrainous features, and exclusion of alternative vestibular diagnoses (Beh, 2022). This framework has substantially improved diagnostic consistency; nonetheless, VM remains underrecognized because patients often present with heterogeneous vestibular complaints, variable attack duration, and incomplete overlap between headache and vestibular symptoms (Smyth et al., 2022; Hac and Gold, 2024).

From a therapeutic perspective, the most difficult clinical question is not whether preventive therapy should be offered, but which agent is most appropriate for a given patient (Villar-Martinez and Goadsby, 2024). In real-world practice, treatment selection is rarely driven by a single symptom or by network meta-analysis rankings alone. Rather, clinicians must account for attack pattern, headache burden, interictal dizziness, emotional distress, visual dependence, functional disability, age, frailty, weight, and cognitive vulnerability (Lempert and von Brevern, 2019; von Brevern and Lempert, 2020). That problem is particularly relevant for venlafaxine and topiramate, two commonly used but biologically and clinically distinct options (Moreno-Ajona, 2026).

This review therefore focuses on a specific and clinically relevant issue: how venlafaxine and topiramate can be selected more rationally in VM when treatment is aligned with phenotype rather than prescribed as interchangeable migraine preventives. The aim is not to propose a rigid algorithm unsupported by evidence, but to synthesize current data into a pragmatic framework that is cautious, guideline-consistent, and applicable to daily practice.

## 2 Methods of literature review

This narrative review was developed from a focused search of PubMed/MEDLINE through January 31, 2026, supplemented by citation tracking of key reviews and consensus papers. Search terms included combinations of “vestibular migraine,” “migrainous vertigo,” “migraine-associated vertigo,” “venlafaxine,” “topiramate,” “persistent postural-perceptual dizziness,” “PPPD,” “prophylaxis,” and “prevention.” Priority was given to clinical studies involving vestibular migraine, randomized or comparative clinical studies, clinically influential reviews, and recent systematic reviews or network meta-analyses evaluating venlafaxine, topiramate, or relevant standard preventive therapies; exclusion criteria comprised non-human studies, small case reports lacking mechanistic insight, and studies not addressing preventive pharmacologic therapy.

Because the objective was interpretive rather than exhaustive, formal risk-of-bias scoring was not performed. The review is therefore intended as a clinically oriented synthesis rather than a systematic review. This distinction is important: the proposed phenotype-guided framework should be understood as a reasoned model derived from available evidence and informed clinical interpretation, not as a validated treatment guideline.

## 3 Why a phenotype-guided approach is needed

VM is broader than episodic spinning vertigo. Patients may report spontaneous vertigo, positional vertigo, rocking, swaying, visually induced dizziness, motion intolerance, head-motion–induced disequilibrium, nausea, phonophobia, photophobia, and interictal sensory discomfort (Baloh, 2020; Li et al., 2019). Moreover, headache may be absent during vestibular attacks, and vestibular symptoms may dominate the clinical picture for years before the diagnosis is considered. This heterogeneity makes a uniform preventive strategy conceptually weak (Staab, 2023).

An equally important source of complexity is overlap with PPPD. PPPD is a chronic functional neuro-otologic disorder characterized by persistent dizziness, unsteadiness, or non-spinning vertigo lasting at least 3 months and exacerbated by upright posture, movement, or complex visual stimuli (Staab et al., 2017). It should be emphasised that PPPD is a distinct diagnostic entity (Barany Society 2017 consensus criteria), rather than a subtype of VM. Contemporary reviews emphasize that PPPD may be precipitated by vestibular migraine, may coexist with VM, and may also be mistaken for VM when episodic features are not carefully explored (Yagi et al., 2024; Trinidade et al., 2023b). In clinical terms, this means that some patients with “VM” are primarily disabled by chronic visually provoked dizziness and affective amplification rather than by discrete vertigo attacks alone.

That distinction matters therapeutically. A patient with recurrent vestibular attacks and substantial migraine-headache burden may reasonably be treated with a conventional migraine preventive (Sharifi et al., 2025). By contrast, a patient with chronic non-spinning dizziness, supermarket intolerance, anxiety, internal motion, and PPPD overlap may require a different treatment emphasis, often involving serotonergic or noradrenergic-serotonergic strategies, vestibular rehabilitation, and broader management of perpetuating factors (Salmito et al., 2017; Sereda et al., 2025). Thus, phenotype-guided prescribing in VM is not an academic exercise; it is an attempt to align treatment with the main drivers of disability.

## 4 Why venlafaxine and topiramate are a useful pair for comparison

Venlafaxine and topiramate are useful to compare because they occupy different therapeutic niches. Venlafaxine is a serotonin-norepinephrine reuptake inhibitor with established efficacy in conventional migraine prophylaxis and potential relevance to patients with anxiety, depressive symptoms, and persistent perceptual dizziness (Ozyalcin et al., 2005; Coutens et al., 2022). Topiramate is a well-established oral migraine preventive with a broader conventional evidence base in headache medicine and a clinical profile that is often attractive in headache-predominant VM (Naegel and Obermann, 2010; Calandre et al., 2024). These two drugs were selected for comparison not because the available evidence suggests they are superior to other drugs such as flunarizine, propranolol and amitriptyline, but because they offer a stark contrast and high complementarity in terms of their mechanisms of action, tolerability profiles and suitability for clinical phenotypes, making them the most appropriate examples to illustrate the concept of ‘phenotype-guided treatment’.

Importantly, the choice between these two agents is not simply a matter of efficacy ranking. It also involves tolerability and fit. Venlafaxine may be advantageous when affective burden, hypervigilance, or PPPD overlap is prominent, but its use may be limited by nausea, insomnia, discontinuation symptoms, or concerns about blood pressure in susceptible patients (Kamp et al., 2024; Hedayat et al., 2022). Topiramate may be appealing when stronger migraine suppression is needed and weight loss would not be problematic, but it may worsen paresthesia, cognitive slowing, appetite suppression, or fatigue, which can be especially relevant in older or cognitively vulnerable patients. These trade-offs make VM an ideal condition in which to apply phenotype-guided thinking (Pearl et al., 2023; Ibrahim et al., 2023; Chen et al., 2024). Table 1 is about studies related to venlafaxine and topiramate for vestibular migraine.

**Table 1:** Key studies informing the use of venlafaxine and topiramate in vestibular migraine.

Study	Design/population	Intervention	Key findings relevant to phenotype-guided selection
Smyth et al. (2022)	Comprehensive practical review	Narrative synthesis of available studies	Evidence base judged low quality overall; treatment recommendations should remain pragmatic and individualized.
Salviz et al. (2016)	Randomized comparative trial in VM	Venlafaxine vs. propranolol	Both groups improved. The study supports venlafaxine as a legitimate VM preventive but does not establish superiority or phenotype specificity.
Liu et al. (2017)	Comparative trial in VM	Venlafaxine vs. flunarizine vs. valproic acid	All groups improved vestibular symptoms; venlafaxine showed benefit in emotional DHI domains and depressive symptoms, supporting use in affective or PPPD-overlap phenotypes.
Gode et al. (2010)	Prospective study in migrainous vertigo	Topiramate 50 mg/day vs. 100 mg/day	Both doses improved vertigo and headache; 50 mg/day appeared more tolerable. Supports topiramate when headache burden is prominent.
Nassar et al. (2023)	Prospective comparative study in VM	Cinnarizine vs. propranolol vs. topiramate	All treatments improved symptoms; topiramate showed favorable headache-related outcomes, reinforcing a role in headache-predominant VM.
Chen et al. (2023)	Network meta-analysis	Randomized evidence only	Venlafaxine showed a favorable efficacy signal in the limited randomized literature; uncertainty remained high because of small sample size and sparse networks.
Almohammed et al. (2025)	Recent systematic reviews	Multiple pharmacologic strategies	Topiramate is used as a first-line preventive treatment for VM, while anti-CGRP monoclonal antibodies may be beneficial for refractory cases.
Vasireddy et al. (2025)	Meta-analyses	Multiple pharmacologic strategies	Later syntheses continued to support individualized use of standard migraine preventives, including topiramate and venlafaxine, but highlighted heterogeneity and lack of phenotype-stratified trials.

Evidence levels were qualitatively categorized as: randomized controlled trials (high–moderate evidence), prospective comparative studies (moderate evidence), systematic reviews/meta-analyses (moderate evidence with heterogeneity), and narrative/pragmatic reviews (low evidence).

## 5 Evidence for venlafaxine in vestibular migraine

The main direct evidence supporting the use of venlafaxine in VM derives from a small yet clinically impactful randomized controlled trial conducted by Salviz et al. (2016). This study compared venlafaxine with propranolol in patients diagnosed with VM, demonstrating that both agents significantly improved vertigo frequency and other dizziness-related outcomes. Notably, no clear superiority of propranolol over venlafaxine was observed. Although the trial was not designed to evaluate phenotype-specific efficacy, it remains one of the few comparative studies that directly inform routine clinical decision-making for VM management.

The second key study is the comparative trial by Liu and colleagues, which evaluated venlafaxine, flunarizine, and valproic acid for VM prophylaxis (Liu et al., 2017). All three treatments improved vestibular symptoms; however, venlafaxine showed a broader signal of benefit across emotional domains of the Dizziness Handicap Inventory and was associated with improvement in depressive symptoms. This finding holds important clinical implications, as it suggests that venlafaxine may provide additional value when VM-related disability extends beyond mere attack frequency to include emotional distress and functional impairment (Furman et al., 2013; Yiannakis et al., 2023).

Direct evidence should also be interpreted in the context of broader migraine prophylaxis literature. Venlafaxine has established efficacy in non-vestibular migraine prevention, which supports its biological plausibility as a preventive option in VM even though extrapolation is imperfect (Roghani et al., 2024; Ceriani et al., 2026). The relevance of this broader literature lies not in the assumption that VM is merely migraine manifesting in the vestibular system, but rather in the shared migraine-related biological mechanisms between VM and non-vestibular migraine, which render standard migraine preventives clinically meaningful in at least a subset of VM patients (Hac and Gold, 2024; Xing et al., 2024).

Taken together, the evidence supports a cautious but favorable view of venlafaxine in at least three VM scenarios (Lauritsen and Marmura, 2017). First, venlafaxine may be a reasonable consideration when psychiatric comorbidity is prominent, especially anxiety or depressive symptoms (May et al., 2024). Second, it may be clinically coherent when visual dependence, chronic non-spinning dizziness, or PPPD overlap suggests that emotional-perceptual factors are contributing substantially to ongoing disability (Webster et al., 2023b). Third, venlafaxine may be clinically advantageous when the therapeutic objective includes both attack reduction and improvement in dizziness-related handicap rather than reduction of headache days alone (Gao et al., 2025).

At the same time, the evidence is insufficient to support indiscriminate use (Hedayat et al., 2022; Robblee et al., 2026). Venlafaxine should be regarded as a rational, phenotype-compatible option rather than a universally preferred first-line agent. Furthermore, the inference that venlafaxine may be beneficial in patients with VM-PPPD overlap or in those with a visual-dependent presentation is primarily derived from indirect evidence in the fields of PPPD and anxiety/depression, as well as the known mechanisms by which SNRI-class drugs modulate the spectrum of mood and perceptual symptoms. Therefore, the above recommendations should be regarded as clinical reasoning rather than definitive evidence. Venlafaxine should be regarded as a rational, phenotype-compatible preventive option rather than a universally preferred first-line agent.

## 6 Evidence for topiramate in vestibular migraine

Topiramate occupies a distinct position in the therapeutic landscape of VM. Its role in conventional migraine prevention is well established, and this extensive body of literature on headache has contributed to its widespread off-label use in VM (Pearl et al., 2023; Choi et al., 2026). In vestibular-specific research, the most frequently cited prospective study is the clinical assessment by Gode and colleagues (Gode et al., 2010), who reported that topiramate reduced both vertigo and headache frequency and severity in patients with migrainous vertigo. In that study, both 50 mg/day and 100 mg/day were effective, but the lower dose appeared more favorable from a tolerability perspective.

More recently, Nassar et al. (2023) conducted a prospective study comparing the efficacy of cinnarizine, propranolol, and topiramate in VM. All three agents improved VM symptoms, but topiramate demonstrated stronger effects on headache-related outcomes and performed favorably across several migraine-specific endpoints. While this study was small and its findings should not be overinterpreted, it reinforces the clinical intuition that topiramate may be particularly useful in patients with VM characterized by a strong headache-predominant phenotype.

Observational evidence further supports topiramate as one of the most commonly effective prophylactic agents in routine clinical practice for VM, although it has not been proven to be superior to other drug classes (Ran et al., 2025). From a practical standpoint, this makes topiramate a reasonable consideration when the clinician regards VM primarily as a migraine-spectrum disorder with vestibular expression. Potential candidates may include patients with frequent migrainous headaches, marked photophobia or phonophobia, prominent sensory hypersensitivity, or a clear need for conventional migraine suppression (Ibrahim et al., 2023; Vélez-Jiménez et al., 2025). However, there are currently no prospective, stratified studies directly comparing the efficacy of topiramate and venlafaxine in VM with a predominant headache pattern. Therefore, this recommendation should be regarded as an extrapolation from the migraine literature rather than VM-specific validation.

However, topiramate has notable adverse effects, including cognitive slowing, paresthesia, appetite suppression, and weight loss (Lampl et al., 2023). These are particularly problematic in VM patients who already experience brain fog or fatigue. In frail or older patients, topiramate may worsen functional status despite reducing attacks (Mistry et al., 2023), suggesting phenotypic mismatch in some cases.

## 7 What recent evidence synthesis adds

The most critical conclusion from recent evidence syntheses is not that a single drug has emerged as optimal, but that the evidence base remains limited and heterogeneous (Smyth et al., 2022). Subsequent quantitative syntheses have reached broadly consistent conclusions, with minor differences in intervention rankings.

The 2023 network meta-analysis by Chen et al. (2023) suggested that valproate, propranolol, and venlafaxine may have favorable efficacy signals for VM, whereas evidence for topiramate was less robust in the randomized subset included in that analysis. By contrast, another 2023 network meta-analysis of randomized clinical trials identified benefit for some established preventives but also highlighted poor tolerability and very limited head-to-head evidence (Chu et al., 2023). More recent systematic reviews published in 2025 concluded that topiramate remains a reasonable first-line or early-line option for VM, and that propranolol, venlafaxine, and other standard migraine preventives continue to play important roles in clinical practice. However, all these conclusions are constrained by substantial heterogeneity in case definitions, intervention protocols, and follow-up durations across included studies (Almohammed et al., 2025; Vasireddy et al., 2025).

These syntheses do not invalidate phenotype-guided practice; rather, they strengthen the case for caution. They show that average treatment effects are uncertain and that pooled estimates cannot answer the clinically crucial question: which patient should receive which preventive? That unanswered question is precisely where a phenotype-guided framework becomes useful, provided it is presented as an informed clinical model rather than as a definitive evidence-based algorithm.

## 8 A pragmatic phenotype-guided framework

Based on current evidence, venlafaxine and topiramate should not be considered interchangeable. Instead, their utility can be contextualized along a spectrum of VM phenotypes, allowing for more personalized treatment selection. Furthermore, PPPD overlap should not simply be regarded as a subtype of VM, nor should it automatically be considered an indication for the use of venlafaxine. It should be clearly noted that the four phenotypes proposed below are not derived from direct evidence from stratified trials, but rather represent a hypothesis-generating framework based on signals of efficacy from the limited existing literature, the known pharmacological mechanisms of the two drugs, and clinical reasoning. Table 2 presents the key points for selecting venlafaxine and topiramate based on practical phenotype guidance.

**Table 2:** Pragmatic phenotype-guided framework for choosing between venlafaxine and topiramate.

Phenotype	Typical clinical clues	Drug more often considered as an illustrative option	Why this choice is coherent	Main cautions
Emotional-perceptual / PPPD-overlap phenotype	Chronic non-spinning dizziness, visual dependence, supermarket or screen intolerance, anxiety, marked DHI burden	Venlafaxine	May address VM prevention while also fitting the emotional-perceptual symptom complex common in PPPD overlap	Use caution in patients with problematic hypertension, activating side effects, or high concern for discontinuation symptoms
Headache-predominant migraine phenotype	Frequent migrainous headaches, strong photophobia / phonophobia, sensory hypersensitivity, clear migraine clustering	Topiramate	May be more aligned with conventional migraine suppression and frequently useful when headache burden drives disability	Use caution in low body weight, frailty, baseline cognitive complaints, or occupations highly sensitive to cognitive slowing
Mixed episodic-plus-interictal phenotype	Recurrent attacks plus persistent residual dizziness	Either, depending on the dominant driver of disability	Venlafaxine may be a reasonable consideration if visual-perceptual symptoms and anxiety dominate; topiramate may be more appropriate if headache and stereotyped migrainous attacks dominate	Medication alone is often insufficient; consider vestibular rehabilitation, and reserve sequential optimization or cautious combination pharmacotherapy for selected specialist settings
Older multimorbid phenotype	Polypharmacy, gait vulnerability, frailty, mild cognitive symptoms, body-weight concerns	Individualized rather than default	Best choice likely depends on global functional fit rather than nominal efficacy alone	Topiramate may worsen cognition or appetite; venlafaxine may be less attractive with uncontrolled blood pressure or autonomic sensitivity

This framework is synthesized from limited evidence, pharmacological plausibility, and clinical reasoning. It should be regarded as a hypothesis-generating tool rather than a validated treatment algorithm.

The emotional-perceptual/PPPD-overlap phenotype is characterized by chronic rocking, visual dizziness, supermarket/screen intolerance, and marked interictal handicap, with disability amplified by perceptual symptoms and affective comorbidity. Here, venlafaxine might be worth considering as one potential approach, especially as it appears to address both migraine pathways and the emotional-perceptual complex (Staab, 2020; Popkirov et al., 2018; Trinidade et al., 2023a).

A second phenotype is the headache-predominant migraine phenotype. These patients have more traditional migraine features, frequent headache days, pronounced photophobia or phonophobia, and vestibular episodes that seem to cluster with clearly migrainous attacks. Topiramate may often be a reasonable consideration here because its clinical identity is that of a conventional migraine preventive, and vestibular improvement is frequently observed alongside headache reduction (Giri et al., 2023; Pellesi et al., 2024).

A third phenotype is the mixed or interictal chronic dizziness phenotype. These patients present with both recurrent attacks and ongoing disequilibrium. Either agent may be reasonable, but the choice depends on what is driving disability at the time of assessment. If residual dizziness is largely visual, perceptual, and anxiety-amplified, venlafaxine may be more congruent. If chronic symptoms coexist with high headache burden and stereotyped migrainous attacks, topiramate may be more appropriate (Qaseem et al., 2025; Naghdi et al., 2023). In both scenarios, medication alone is often insufficient; vestibular rehabilitation, trigger management, and education remain important adjuncts.

A fourth phenotype is the older multimorbid patient. In this group, apparent treatment efficacy can be offset by impaired tolerability. Topiramate may worsen cognition, appetite, or gait confidence, whereas venlafaxine may be less attractive in patients with poorly controlled hypertension, pronounced autonomic symptoms, or complex polypharmacy. For older patients, the best preventive is not simply the one with the highest theoretical efficacy, but the one that improves function without creating new disability. This group is where geriatric fit matters most, and where phenotype-guided selection becomes especially valuable (Starling, 2018; Paparella et al., 2025).

Pragmatically, clinicians should base treatment decisions on at least six key domains (Sarna et al., 2021): (1) the predominance of headache symptoms; (2) the presence of emotional or psychiatric comorbidities; (3) overlap with PPPD or visually induced dizziness; (4) body weight considerations; (5) cognitive vulnerability; and (6) the patient’s prioritized treatment outcome. The preferred endpoint may include fewer vertigo attacks, reduced interictal dizziness, improved confidence in visually complex environments, fewer headache days, or enhanced overall function. Explicitly identifying this target before prescribing can improve both the selection of the appropriate agent and the interpretation of treatment response.

## 9 Practical treatment considerations

Treatment should generally begin with a low dose and slow titration, especially in patients with chronic dizziness, medication sensitivity, or high anxiety (Sharon et al., 2024). This strategy is particularly important for venlafaxine and topiramate to improve tolerability and minimize adverse effects and is consistent with established principles of good clinical practice (Arslan et al., 2026; Webster et al., 2023a).

Response assessment should extend beyond attack counts. The most useful follow-up questions are often phenotype-specific: Are attacks less frequent? Is visually induced dizziness better? Is interictal disequilibrium improved? Is the patient less avoidant of movement or public environments? Are headache days fewer? Has the emotional burden of dizziness improved? Such questions are more informative than a single generic dizziness score and more consistent with the logic of individualized care.

Combination care is frequently necessary. In PPPD-overlap phenotypes, medication is often best paired with vestibular rehabilitation and targeted education about symptom mechanisms (Dao Pei et al., 2025). It is particularly important to emphasise that, before considering the use of venlafaxine in patients with so-called ‘VM-PPPD overlap’, clinicians must carry out a structured diagnostic assessment to confirm whether a diagnosis of PPPD is indeed valid. In headache-dominant phenotypes, attention to migraine triggers, sleep regulation, and rescue medication strategy remains important (Li et al., 2025). Pharmacological combination prophylaxis may ultimately prove useful in selected refractory or mixed-phenotype patients, but direct VM-specific evidence remains insufficient, and current migraine guidance advises caution with oral preventive combinations because of drug–drug interactions and tolerability concerns (Baron and Steenerson, 2025). Therefore, phenotype-guided prescribing should be understood as one component of phenotype-guided management, not as a replacement for multimodal care.

Multimodal management is essential in VM, especially in patients with PPPD overlap, where pharmacotherapy is only one element of a comprehensive strategy. Non-pharmacological interventions, including vestibular rehabilitation, graded exposure, avoidance reduction, psychologically informed rehabilitation such as CBT and mindfulness, sleep optimization, regular aerobic exercise, migraine lifestyle modification, and patient education, are often critical to functional recovery. In headache-predominant VM, trigger management and lifestyle adjustments are particularly important, whereas in older multimorbid patients, rehabilitation and exercise should be individualized based on physical tolerance and comorbidities. Pharmacotherapy should always be delivered within this broader multimodal framework rather than in isolation.

## 10 Mechanistic plausibility and biological rationale for phenotype-based drug selection

Current evidence suggests that VM is not a single pathophysiological entity but rather a spectrum of clinical syndromes characterized by the interplay of multiple neural mechanisms. Its core features include central sensory integration dysfunction, activation of the trigeminovascular system, dysregulation of emotional control networks, and a hypersensitive state of the vestibulo-thalamo-cortical pathway (Ceriani, 2024; Ma et al., 2024). This mechanistic heterogeneity provides a biological basis for phenotype-guided selection of preventive medications.

As a serotonin-norepinephrine reuptake inhibitor (SNRI), venlafaxine is hypothesized to exert therapeutic effects via monoaminergic modulation of descending pain pathways and by reducing hypervigilance, threat perception, and pathological visual dependence-features prominent in VM with PPPD overlap (Shahid et al., 2025). This mechanism provides a biological rationale for the use of venlafaxine in patients with this co-occurring condition. However, it must be emphasised that this rationale alone cannot replace a careful diagnostic assessment; before considering venlafaxine, it is essential to confirm that a diagnosis of PPPD has indeed been established. A randomized controlled trial in VM patients showed venlafaxine significantly improved Vertigo Symptom Scale (VSS) and Dizziness Handicap Inventory (DHI) emotional subscale scores, suggesting possible benefits for emotional and functional disability (Liu et al., 2017). Systematic reviews and meta-analyses confirm SNRIs are superior to placebo for reducing VM-related dizziness burden, with greater benefits in patients with comorbid anxiety or depression (Wang et al., 2020). In contrast, topiramate primarily acts on cortical hyperexcitability and sensory hypersensitivity via multi-target mechanisms: inhibition of voltage-gated sodium channels, suppression of glutamatergic transmission, and enhancement of GABAergic inhibition (Andreou and Goadsby, 2011; Hoffmann et al., 2014).

Notably, while network systematic reviews and meta-analyses identify both agents as effective VM preventives, existing comparative studies are limited by small sample sizes, heterogeneous diagnostic criteria, and inconsistent outcomes, precluding conclusions on therapeutic equivalence (Byun et al., 2021). Thus, the two drugs are not interchangeable; each may be more coherent for VM subtypes hypothetically driven by distinct pathophysiology. Venlafaxine may be reasonably considered for patients with comorbid anxiety/depression, VM-PPPD overlap, visually induced dizziness, or significant emotional/functional impairment (Eggers and Staab, 2024; Wang et al., 2020). Topiramate may be more suitable for headache-predominant VM, marked sensory hypersensitivity, or when classic migraine suppression is the primary goal (with acceptable cognitive/metabolic tolerability) (Tinsley and Rothrock, 2021; Vécsei et al., 2015). Sequential optimization or cautious low-dose combination (under specialist supervision) warrants investigation in mixed phenotypes, high disease burden, or monotherapy non-response (Luo et al., 2012; Gomez-Lumbreras et al., 2026).

In summary, the potential benefit of venlafaxine in VM-PPPD overlap and the possible advantage of topiramate in headache-predominant VM are biologically and pathophysiologically plausible. However, it must be re-emphasized that these mechanistic inferences have not yet been validated in prospective clinical trials. The translation of mechanistic plausibility into clinical efficacy prediction requires confirmation through well-designed phenotype-stratified comparative trials. Until such evidence becomes available, the above mechanistic discussion should be regarded as background knowledge to support individualized clinical reasoning rather than as confirmatory evidence guiding specific treatment decisions.

## 11 Limitations and future directions

Several limitations constrain strong conclusions. Firstly, the fact that this study did not include databases such as Embase and the Cochrane Library constitutes a limitation of this study. Secondly, direct comparative data on venlafaxine and topiramate in VM are sparse. Many studies mix definite and probable VM or rely on older labels such as migrainous vertigo, limiting comparability. Commonly used outcomes (vertigo frequency, DHI, headache measures) do not fully capture perceptual symptoms, visual dependence, or functional recovery. All recommendations regarding the combination of VM-PPPD with venlafaxine, and of headache-dominant VM-PPPD with topiramate, are based on *post hoc* inferences and indirect evidence. Whilst these inferences are biologically plausible, they have not yet been confirmed by prospective phenotypic stratification trials. Few studies explicitly characterize psychiatric comorbidity or PPPD overlap, though these likely modify treatment response (Fu et al., 2025; Ferlito et al., 2026). Thus, our proposed framework is partly inferential-reflecting the current state of the field, which supports individualized reasoning but not a definitive algorithm.

Future advances depend on matching treatments to patient phenotypes. Studies should separate definite from probable VM, prespecify clinical phenotypes, and document PPPD overlap. Standardized endpoints are essential (Joffily et al., 2025). Beyond attack frequency, future trials should capture visual dependence, interictal dizziness, quality of life, treatment persistence, and patient-reported functional recovery. In addition, treatment protocols optimised on the basis of biomarkers or clinical characteristics should be adopted. Emerging treatment strategies include CGRP monoclonal antibodies, Gepants and neurostimulation. Although these treatments have demonstrated some efficacy in migraine, evidence regarding their use in vestibular migraine remains limited, and no stratification studies based on clinical phenotypes have yet been conducted. Future research should focus on conducting prospective, stratified clinical trials and integrating vestibular function, neuropsychiatric factors and migraine mechanisms to establish a more precise, personalised treatment system.

## 12 Conclusion

In summary, venlafaxine and topiramate should not be considered therapeutically interchangeable in VM. Instead, they appear to address distinct yet partially overlapping clinical phenotypes within the VM spectrum. Venlafaxine may be more coherently considered for emotionally amplified presentations, particularly those overlapping with PPPD or characterized by visually induced dizziness. In contrast, based on the available evidence and mechanistic reasoning, topiramate may offer greater benefit in headache-dominant phenotypes and those driven by sensory hypersensitivity.

Given the persistent heterogeneity of available evidence, the most clinically defensible strategy remains individualized, phenotype-guided prescribing, grounded in symptom architecture, comorbidity burden, functional impairment, and tolerability considerations. No VM-specific trial currently supports venlafaxine-topiramate combination therapy; therefore, such an approach should be regarded as hypothesis-generating rather than standard practice. Treatment success should be judged not only by symptom reduction but also by restoration of function. Future prospective, phenotype-stratified comparative trials are essential to advance precision prophylaxis in VM.
